# Expression of Drosophila Adenosine Deaminase in Immune Cells during Inflammatory Response

**DOI:** 10.1371/journal.pone.0017741

**Published:** 2011-03-11

**Authors:** Milena Novakova, Tomas Dolezal

**Affiliations:** Department of Molecular Biology, Faculty of Science, University of South Bohemia, Ceske Budejovice, Czech Republic; Buck Institute for Age Research, United States of America

## Abstract

Extra-cellular adenosine is an important regulator of inflammatory responses. It is generated from released ATP by a cascade of ectoenzymes and degraded by adenosine deaminase (ADA). There are two types of enzymes with ADA activity: ADA1 and ADGF/ADA2. ADA2 activity originates from macrophages and dendritic cells and is associated with inflammatory responses in humans and rats. Drosophila possesses a family of six ADGF proteins with ADGF-A being the main regulator of extra-cellular adenosine during larval stages. Herein we present the generation of a GFP reporter for ADGF-A expression by a precise replacement of the *ADGF-A* coding sequence with *GFP* using homologous recombination. We show that the reporter is specifically expressed in aggregating hemocytes (Drosophila immune cells) forming melanotic capsules; a characteristic of inflammatory response. Our vital reporter thus confirms ADA expression in sites of inflammation in vivo and demonstrates that the requirement for ADA activity during inflammatory response is evolutionary conserved from insects to vertebrates. Our results also suggest that ADA activity is achieved specifically within sites of inflammation by an uncharacterized post-transcriptional regulation based mechanism. Utilizing various mutants that induce melanotic capsule formation and also a real immune challenge provided by parasitic wasps, we show that the acute expression of the ADGF-A protein is not driven by one specific signaling cascade but is rather associated with the behavior of immune cells during the general inflammatory response. Connecting the exclusive expression of ADGF-A within sites of inflammation, as presented here, with the release of energy stores when the ADGF-A activity is absent, suggests that extra-cellular adenosine may function as a signal for energy allocation during immune response and that ADGF-A/ADA2 expression in such sites of inflammation may regulate this role.

## Introduction

Extra-cellular adenosine is an important regulatory molecule with a low physiological concentration that can rapidly increase during tissue damage, inflammation, ischemia or hypoxia. In damaged tissues and inflammatory responses, extra-cellular adenosine is a product of ATP degradation, mediated by a cascade of ectoenzymes. Both ATP and adenosine stimulate purinergic receptors, and their release, signaling and progressive decrease regulate the onset of the acute inflammatory response, the fine-tuning of ongoing inflammation and its eventual down-regulation [1]. However the overall regulation of inflammatory responses by ATP and adenosine is quite complex, especially in mammalian adaptive immunity, and thus many questions regarding the roles of ATP and adenosine persist.

An important step in this regulation is the degradation of adenosine by adenosine deaminase (ADA), an enzyme that converts adenosine and deoxyadenosine to inosine and deoxyinosine, respectively. Generally, two types of enzymes with the ADA activity are known; the ADA1 family proteins and adenosine deaminase-related growth factors (ADGF) or ADA2-like proteins. ADA1 is present both in prokaryotes and eukaryotes and has been studied for long time, mainly because its deficiency in humans causes severe combined immunodeficiency (SCID) syndrome. Although the most studied function of ADA1 is the reduction of toxic intracellular levels of adenosine, especially for lymphocytes [2], ADA1 has also been detected as an ectoenzyme associated with cell surface receptors [3].

Although ADA2 activity in human plasma was discovered long time ago [4], the protein responsible for this activity was only recently isolated [5]. ADA2 (ENSG00000093072) is a secreted protein and adenosine seems to be its only substrate [6]. ADA2 activity is significantly increased in the pleural effusions of tuberculosis patients [7] and the serum of patients infected with HIV [8]. It was also shown that macrophages are the source of ADA2 activity during inflammatory responses in rats [9]. Zavialov *et al.* [10] showed that ADA2 protein secreted by monocytes undergoing differentiation is the only source of ADA activity from these cells. They further revealed that human ADA2 promotes CD4^+^ T cell-dependent differentiation of monocytes to macrophages and their subsequent proliferation and that this role of ADA2 is independent of its ADA catalytic function.

ADA2 belongs to the family of ADGF proteins, first characterized in insect [11]. The ADGF-A protein (FBgn0036752) from *Drosophila melanogaster* is similar to secreted human ADA2 [12] and both proteins share all structure domains, including those considered unique to ADA2 [13]. However, ADGF-A, as with other ADGFs from lower species, has a higher affinity for adenosine (similar to ADA1) than human ADA2. According to Zavialov *et al.* [13], human ADA2 may have become specialized during evolution to be an adenosine deaminase specifically active in sites of high adenosine concentration and lower pH, typified by sites of inflammation.

We have showed that *ADGF-A* mRNA is expressed in the Drosophila hematopoietic organ, called the lymph gland [12] and that this expression is required for larval survival [14]. Drosophila hematopoiesis and cellular immunity are much simpler than in vertebrates, nevertheless both systems share many features [15]. The main component of cellular immunity in flies is represented by plasmatocytes that are macrophage-like cells with phagocytic activity. These cells are responsible for the inflammatory response to tissue damage (clearance of tissue debris and healing) and infection. In similarity to vertebrate systems, these macrophage-like cells are attracted to sites of injury where they adhere and become phagocytic [16]. This ability to recognize and adhere to damaged or “nonself” tissue is an ancestral feature of blood cells. In the case of larger objects, such as parasitic wasp eggs, specialized cells called lamellocytes (large flat cells) differentiate from prohemocytes and encapsulate the foreign object, thus isolating it from the rest of the body cavity. The intruding object is then destroyed by melanization; an important immune mechanism in arthropods utilizing toxic quinone substances and other short-lived reaction intermediates [17]. These substances are also involved in the formation of more long-lasting products such as the melanin that physically encapsulates pathogens. Furthermore, reaction intermediates in the melanin pathway participate in the wound healing process by the formation of covalent links in damaged tissues and results in sclerotization.

Since hemocyte *ADGF-A* mRNA expression is required for larval survival, we were interested in the regulation of its expression. However, because there is no available antibody against ADGF-A, we decided to produce a vital GFP reporter for its expression using homologous recombination. Here we show that this reporter is expressed in vivo, in aggregating larval hemocytes at sites of inflammation and that the acute expression of the ADGF-A protein is most probably regulated at post-transcriptional level.

## Materials and Methods

### Drosophila strains and culture

The AGFP reporter was produced as described in File S1. The line depicted as *AGFP[23]*, was used for the experiments presented herein; this line was kept as *w; AGFP[23]/TM6B* and *w; AGFP[23]/TM3 Act>GFP Ser* and crossed to the wild-type *Oregon R* strain to obtain *AGFP[23]/+*. Additional lines used in this work were as following: *Oregon-R* as wild-type strain, *w; adgf-a^karel^/TM6B*, *w; cactus[E8]/CyOGFP*; *AGFP[23]/TM6B*, *w; cactus[IIIG]/CyOGFP*, *w; cactus[D13]/CyOGFP* and *y[1] v[1] hop[Tum]/FM7c*. All flies were raised on corn meal/sucrose/agar medium and kept at 25°C.

### Wasp parazitation

*w; AGFP[23]/TM6B* flies crossed to *Oregon-R* flies and only *Oregon-R* flies were left to lay eggs for 12 hours in regular food-containing vials. The second-instar larvae were then immunized by a parasitic wasp *Leptopilina boulardi* for two hours. The wasps were then discarded and the infected larvae permitted to continue their development for another 24–48 hours at 25°C. GFP fluorescence was then analyzed in encapsulated wasp eggs dissected from the third-instar larvae, as described below.

### GFP fluorescence analysis

GFP fluorescence was analyzed in dechorionated embryos, whole and dissected larvae and pupae and in adult flies using fluorescent either stereomicroscopy and inverted microscopy. Hemocytes, melanotic capsules and parasitic wasp eggs were obtained by careful dissection of third-instar larvae in a drop of Ringer solution on a microscopic slide and immediately examined by one of fluorescent microscopic techniques. Samples were analyzed using differential interference contrast (DIC) and the Olympus U-MWG2 GFP filter settings. Micrographs were obtained using a color CCD, Olympus DP70 camera.

### Expression analysis by RT-PCR and Real-time PCR

Total RNA was isolated using TRI Reagent (Molecular Research Center, Cincinnati, Ohio) according to manufacturer's instructions. RNA was isolated from *w; AGFP[23]/TM3 Act>GFP Ser* stock in the following hours after egg laying: 14–18 h (embryos), 34–38 h (first-instar larvae), 58–62 h (second-instar larvae), 82–86 h (third-instar larvae) and from the third-instar wandering larvae, white prepupae, pupae 26–34 h after pupation and three days-old adult males and females. cDNA was generated from 2.4 µg of RNA treated with TURBO DNase I (Ambion, Austin, TX) using the SuperScript III Reverse Transcriptase kit (Invitrogen, Carlsbad, CA) with oligo(dT) primering. *Actin* amplification was used to determine the final dilution of cDNA from each sample for the PCR reactions which were performed using the following primers: 5′-TACCCCATTGAGCACGGTAT-3′ and 5′-GGTCATCTTCTCACGGTTGG-3′ for *Actin*, 5′-AATCGGAGCTCCTCAATCGC-3′ and 5′-GCTACACATTGATCCTAGC-3′ for *AGFP*, 5′-AGGTTCTCATCCACAGTGG-3′ and 5′-CGGACTACTACTACAAAGC-3′ for *ADGF-A*.

Third-instar wandering larvae of the following genotypes were used for Real-time PCR analysis: *AGFP[23]/+*, *AGFP[23]/AGFP[23]*, *cactus[IIIG]/cactus[E8]*; *AGFP[23]/+*, *cactus[D13]/cactus[E8]*; *AGFP[23]/+*. Total RNA was isolated (1) from 60 whole larvae using TRI Reagent and (2) from hemocytes as follows: 15–30 larvae were dissected one at a time in Ringer solution in 1.5-ml tubes and the hemolymph collected (the remaining body was discarded). The hemolymph were then centrifuged for 5 min at 1500×g, the supernatant discarded and total RNA isolated from the collected hemocytes using the RNAqueous-Micro kit (Ambion, Austin, TX). The RNA was then treated with TURBO DNase I. The concentration of RNA was determined by NanoDrop ND1000 (Thermo Fisher Scientific Inc., Waltham, MA) analysis and cDNA synthesized using the SuperScript III Reverse Transcriptase kit with oligo(dT) primering. The cDNA was then treated with RNaseH (Takara, Japan). Samples were analyzed by Real-time PCR using 1× Syber green Supermix (Biorad, Hercules, CA) and 5 pmol of each primer in CFX96 Real Time System C1000 (Biorad) as triplicate measurements. The following primers were used: 5′-CTTCATCCGCCACCAGTC-3′ and 5′-CACGTTGTGCACCAGGAA-3′ for *Rp49*, 5′-GGATCCCCCAGTCAACGG-3′ specific for *ADGF-A*, 5′-TGCTTCTGCTAGGATCAATGTGTA-3′ specific for *AGFP* and 5′-CTGAGTGGATGCGAATGAGAGTG-3′ common for *ADGF* and *AGFP*. Biorad CFX Manager software was used to quantify transcript levels by comparison to relative standard curves generated for each gene by serial (5×) dilutions of Drosophila genomic DNA. Levels of *ADGF-A* and *AGFP* were normalized with *Rp49* levels from the same cDNA samples and plotted as *ADGF-A* or *AGFP* expression level relative to *Rp49*. Results are shown as mean ± SEM of three independent experiments. The experimental significance was determined using a Student's *t* test utilizing the STATISTICA 6 software (StatSoft) package.

## Results

### Generation of the *ADGF-A* reporter system

**Figure 1:**
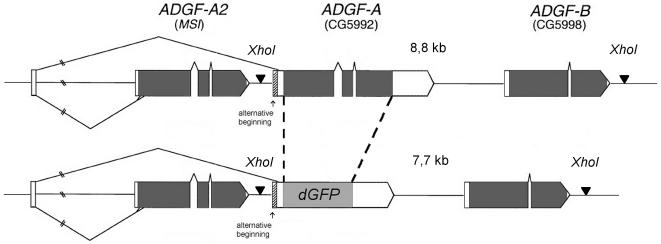
Schematic diagram of the *ADGF-A* coding sequence replacement by the coding sequence of a reporter gene encoding the destabilized GFP. The arrangement of three *ADGF* genes on the wild-type chromosome III is shown on the top. The bottom panel shows gene organization in the reporter system. Coding sequences are depicted by dark boxes and white boxes indicate 5′ and 3′ untranslated regions of the genes. Introns are represented by chevron-shaped lines, intergenic spaces by horizontal lines. Dashed lines depict the replacement. 5′-3′ orientation of all three genes is shown from left to right. The *ADGF-A* gene shares the first exon with the *ADGF-A2* gene but it also uses an alternative transcription start site, specific only for the *ADGF-A* gene marked by an arrow. *Xho*I restriction sites in the sequence are depicted by triangles and the fragment lengths after the digestion are shown above each sequence in kilobases.

We used homologous recombination to produce a GFP reporter for the analysis of endogenous, in vivo, levels of *ADGF-A* expression by the precise replacement of the *ADGF-A* coding sequence with that of *GFP* (Figure 1). This therefore ensured that all the surrounding regulatory sequences, including 5′ and 3′ untranslated regions, of *ADGF-A* locus remained intact. We utilized a destabilized version of the Green Fluorescent Protein (*dGFP*) [18], that makes it possible to analyze dynamic changes in *ADGF-A* expression since the dGFP does not accumulate in cells as it is turned-over in around 2–4 hours.

We used the *‘ends-in’* version of homologous recombination [19] that permitted precise exchange of the coding sequences in two steps and without leaving any traces of the recombination process. The first recombination step produced a duplication in the *ADGF-A* containing region, with the *ADGF-A* coding sequence at one side and the *dGFP* coding sequence on the other side (File S1). The second recombination step resulted in a reduction (File S1) of this configuration, thus producing the sequence assembly as shown in Figure 1. Details of the whole procedure are described in File S1.

**Figure 2:**
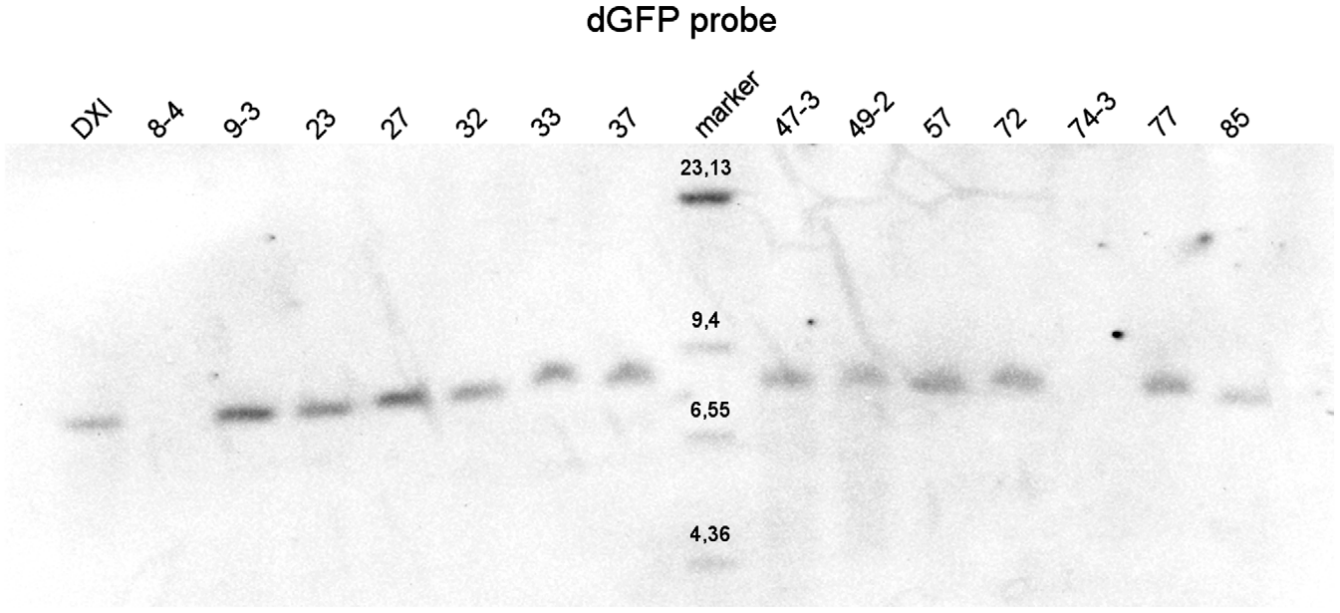
Southern blot analysis of the second recombination step. Genomic DNA was digested with *Xho*I restriction enzyme and the membrane was hybridized with the *dGFP* probe. The size of the *Xho*I fragment hybridised with the *dGFP* probe, after the successful replacement is 7.7 kb (Figure 1 and File S1) - the *DXI* line with duplication was used as a control for the fragment size (File S1). Sizes in kilobases are depicted above each band of the DIG-labeled marker.

We obtained a total 12 lines exhibiting the correct replacement as judged by the successful hybridization of the *dGFP* probe to DNA fragments of correct size in Southern blotting analysis (Figure 2). We named this replacement as *AGFP* (for *ADGF-A GFP* reporter). The Southern blot results were further confirmed by PCR and sequencing (data not shown). In two lines (8-4 and 74-3; Figure 2), the second recombination event occurred downstream of the *ADGF-A*/*dGFP* sequences restoring a wild-type gene arrangement (File S1). This was confirmed by PCR analysis resulting in an amplification of the *ADGF-A* fragment size instead of the shorter *dGFP* sequence (data not shown).

Since the replacement of the *ADGF-A* coding sequence results in a null mutation of this gene we examined the homozygous *AGFP* or heterozygous *adgf-a/AGFP* larvae and found that they exhibited the same phenotype as the originally described *adgf-a* mutant [14] (see further); thus providing confirmation at the phenotypic level that the replacement successfully occurred within the *ADGF-A* locus.

### Expression of *AGFP* mRNA

Our molecular characterization (Figure 2 and File S1) confirmed that the coding sequence of the *ADGF-A* gene had been accurately replaced by the coding sequence of *dGFP*. Therefore, we further analyzed if the expression of *AGFP* reporter mRNA corresponded to the endogenous *ADGF-A* expression.

**Figure 3:**
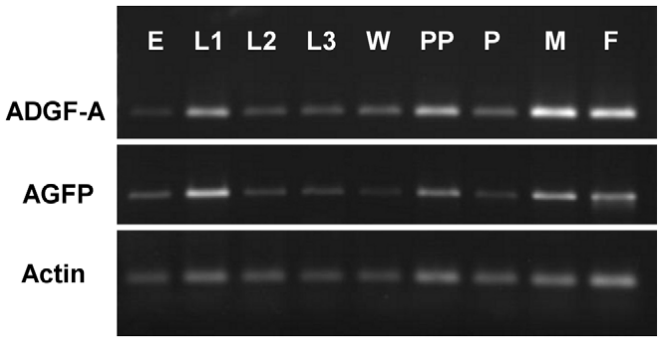
Developmental profile of *ADGF-A* and *AGFP* mRNA expression by RT-PCR. The total RNA was isolated from *w; AGFP[23]/TM3 Act>GFP Ser* individuals at the following stages: embryos (E), first-instar larval stage (L1), second-instar larval stage (L2), third-instar larval stage (L3), wandering larvae (W), prepupae (PP), pupae (P), male adults (M) and female adults (F). RNA was reverse transcribed and amplified by PCR using *ADGF-A* or *AGFP* specific primers. *Actin* cDNA was amplified as template control.

*ADGF-A* mRNA is normally expressed throughout all stages of development [12] (Figure 3) and similarly, the *AGFP* mRNA was also expressed at all stages (Figure 3). This demonstrated that our *AGFP* reporter expression system was able to faithfully report the temporal expression of the endogenous *ADGF-A* gene.

**Figure 4:**
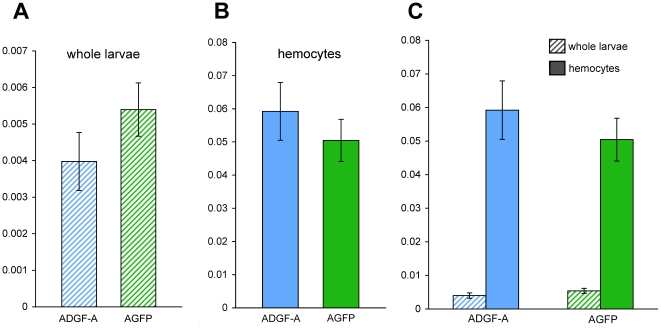
Real-time PCR analysis of the *ADGF-A* and *AGFP* expression in the *AGFP[23]/+* larvae. (A) Expression level in whole larvae and (B) circulating hemocytes. (C) Combined expression in whole larvae and in hemocytes plotted on one graph to demonstrate the difference in levels. Data represent mean values ± SEM, normalized relative to the endogenous control.

Moreover Figure 4A demonstrates that the overall levels of mRNA are similar (p = 0,286) for both *ADGF-A* and *AGFP* transcripts when measured in samples obtained from the *AGFP[23]/+* heterozygous wandering larvae; where *ADGF-A* is expressed from wild-type chromosome and *AGFP* from the modified one.

We had previously shown that the expression of *ADGF-A* in the larval hematopoietic system is crucial for larval survival [14]. In agreement with this observation, Figure 4C demonstrates that the expression of *ADGF-A* in larval hemocytes is ∼10 times stronger compared to overall larval expression. This cell type-specific expression pattern is also represented in the expression of the *AGFP* reporter (Figure 4B, p = 0,344), further confirming the fidelity of the created reporter system in relaying the normal pattern of endogenous *ADGF-A* expression.

### ADGF-A expression analysis by the AGFP reporter

Utilizing our AGFP reporter we analyzed the in vivo *ADGF-A* expression by observing the fluorescence of dGFP in the *AGFP/+* animals at all developmental stages. Surprisingly, we did not detect any fluorescence above background levels in any tissue/cells (data not shown), including larval hemocytes where we could detect mRNA in relatively high quantities. We examined several independent versions of our reporter line, including *AGFP[23]* and *AGFP[72]*, that were analyzed thoroughly (data not shown).

We also tried to detect the dGFP protein in fixed *AGFP/+* embryos and larvae by immuno-fluorescence using an anti-GFP antibody. We hoped this would overcome both the background autofluorescence that is close to the green spectrum (by using secondary antibody conjugated to the red flourophore, Cy3) and potentially low levels of dGFP protein present by signal amplification achievable using a polyclonal antibody. We also tried to detect the dGFP protein in whole larval and hemocyte lysates by western blotting. However, we were unable to detect the dGFP protein by any of these methods (data not shown), suggesting that there is either no expression of the AGFP reporter or that expression levels fall below our detection thresholds.

**Figure 5:**
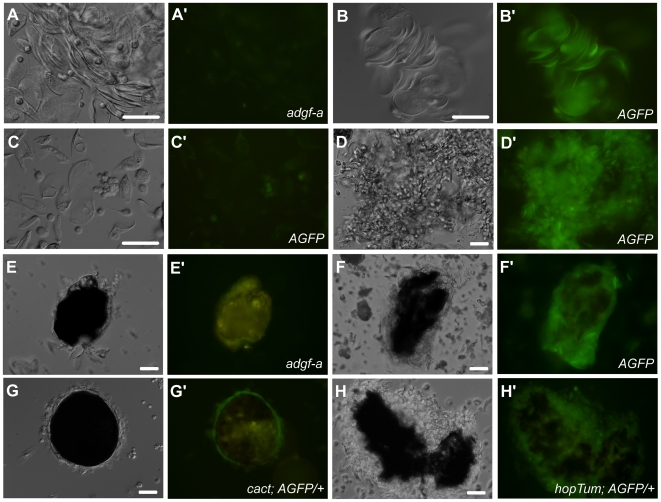
AGFP expression in hemocytes and melanotic capsules obtained from the late third-instar larvae. (A) Aggregating lamellocytes from the *adgf-a* mutant larvae without AGFP as a control to account for autofluorescence. (B) Aggregating lamellocytes from *AGFP[23]* homozygotes showing GFP fluorescence. (C) Non-aggregating plasmatocytes and lamellocytes from *AGFP[23]* homozygotes that rarely displayed any GFP fluorescence. (D) A clump of plasmatocytes and lamellocytes from *AGFP[23]* homozygotes exhibiting strong GFP expression. (E) A melanotic capsule from the *adgf-a* mutant showing only yellowish autofluorescence of the melanized region. (F) GFP fluorescence in hemocytes surrounding a melanotic capsule from *AGFP[23]* homozygotes. (G) A melanotic capsule from the null *cactus* mutant with AGFP reporter (*cactus[E8]/cactus[D13]*; *AGFP[23]/+*) showing GFP expression in surface lamellocytes. (H) A melanizing clump with lamellocytes and plasmatocytes expressing GFP in *hop^Tum^*; *AGFP[23]/+* mutant. Scale bar (50 µm) is shown in white on DIC images. Genotypes are shown on corresponding fluorescent micrographs distinguished with an apostrophe. All fluorescent images were captured using the same exposure settings.

Interestingly, we did detect GFP fluorescence in aggregating hemocytes of *AGFP* homozygous animals (Figure 5B,D,F). *AGFP* homozygotes are effectively *adgf-a* gene mutants and thus present the same mutant phenotype that includes melanotic capsule formation associated with differentiating hemocytes [14]. The strongest GFP expression was detected in the melanotic capsules (Figure 5F). These capsules are formed by aggregating plasmatocytes and lamellocytes, with inside melanization. While most of the circulating, *i.e.* non-adhered plasmatocytes and lamellocytes did not express GFP (Figure 5C), the aggregating cells did (Figure 5B). This was especially apparent in large aggregates (Figure 5D) that eventually formed melanized capsules (Figure 5F).

**Figure 6:**
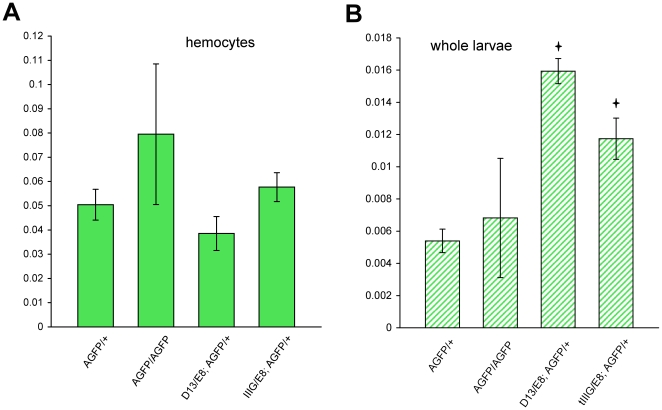
Real-time PCR analysis of *AGFP* mRNA expression. (A) Expression in hemocytes and (B) in wandering larvae. Data represent the mean values ± SEM, normalized relative to the endogenous control. The following genotypes are shown: *AGFP[23]/+*, *AGFP[23]/AGFP[23]*, *cactus[D13]*/*cactus[E8]*; *AGFP[23]/+* and *cactus[IIIG]*/*cactus[E8]*; *AGFP[23]/+*. Columns indicated by asterisks are significantly different from *AGFP[23]/+* control (hemocytes: p(*AGFP/AGFP*) = 0,45; p(*D13/E8*) = 0,27; p(*IIIG/E8*) = 0,45; larvae: p(*AGFP/AGFP*) = 0,2862; p(*D13/E8*) = 0,00028; p(*IIIG/E8*) = 0,0063).

It was possible that this observed fluorescence was due to an increased expression of *AGFP* mRNA in *adgf-a* deficient, *AGFP* homozygous larvae. Therefore we measured *AGFP* mRNA levels in *AGFP* homozygous and heterozygous larvae and hemocytes. We did not detect any significant differences in mRNA levels (P = 0,286 for larvae and p = 0,45 for hemocytes, respectively) between homozygotes and heterozygotes (heterozygotes levels were doubled due to a presence of only one *AGFP* allele) (Figure 6A,B). It is important to note that there was quite high variability in the mRNA levels of homozygotes most likely attributable to variability in the mutant phenotype. This is because development was quite delayed making it difficult to collect larvae at precisely the same stage. More valuable results for comparison were obtained using *cactus* mutants (see below).

### AGFP expression in *cactus* mutants

The melanotic capsule phenotype of the *adgf-a* mutant is similar to another phenotype caused by the constitutive activation of Toll signaling [20]. This can be achieved by a zygotic mutation in the *Cactus* gene (FBgn0000250), an inhibitor of the Rel transcription factors *Dorsal* and *Dif*, that activate Toll target genes. Therefore we tested if *cactus* mutations would lead to the expression of our *AGFP* reporter.

Similarly to *AGFP* homozygous larvae, both hypomorphic (*cactus[E8]*/*cactus[IIIG]*) and null (*cactus[E8]*/*cactus[D13]*) mutant combinations [20] together with *AGFP[23]/+* showed GFP fluorescence in aggregating hemocytes and especially in the outer border of melanotic capsules (data not shown and Figure 5G). Since this result was obtained in *AGFP[23]/+* heterozygotes, it demonstrated that it was not the *adgf-a* mutant phenotype, nor the presence of two *AGFP* copies that are required for AGFP reporter expression. Rather it was the behavior of hemocytes and particularly their aggregation that causes the expression of dGFP protein.

We also assayed the *AGFP* mRNA levels in the *cactus* mutant background. We did not detect significant changes in *AGFP* mRNA expression when comparing hemocytes with and without *cactus* mutations (Figure 6A). When the *AGFP* levels were compared in whole larvae, the amount was however increased in the *cactus* mutants (Figure 6B). Since we did not observe GFP expression in any other cells/tissues of the *cactus* mutants, besides the hemocytes, this difference was most likely caused by the increased number of hemocytes per larva in the *cactus* mutants [20].

### AGFP expression in *hop^Tum^* mutant

The *hop^Tum^* mutation (FBal0005547) results in the hyperactivity of the Jak kinase Hopscotch [21], leading to the aggregation of hemocytes and the formation of melanotic capsules. In similarity to *AGFP* homozygotes and *cactus* mutants, *hop^Tum^* mutants also promote the expression of the AGFP reporter in hemocyte aggregates that were forming melanotic capsules (Figure 5H).

### AGFP expression in larvae infected by parasitic wasps

**Figure 7:**
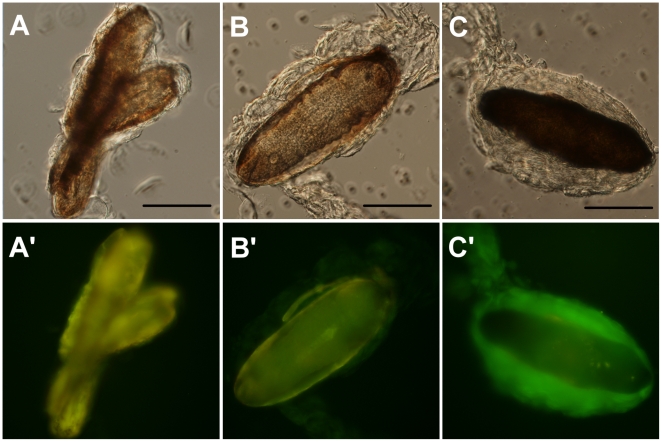
Encapsulation of parasitic wasp egg. (A) Partially encapsulated wasp eggs from *Oregon-R* larva as a control displaying only yellow auto-fluorescence of the wasp eggs. (B) Partially encapsulated wasp egg from *AGFP[23]/+* larva showing a faint but clearly visible green fluorescence of AGFP reporter in the encapsulating lamellocytes. (C) A fully encapsulated and melanized wasp egg from *AGFP[23]/+* larva exhibitting strong AGFP expression in the encapsulating material. DIC (top with 100-µm black scale bar) and corresponding fluorescence (bottom labeled by letter plus an apostrophe) images are shown in each panel.

As we know that the AGFP reporter was expressed in larvae harboring different mutations that induced melanotic capsule formation, we used a parasitic wasp *Leptopilina boulardi* to test its expression during a real immune challenge that would result in melanotic capsule formation. When the parasitic wasps lay their eggs in Drosophila larvae, the plasmatocytes attach themselves to the surface of the egg and thereafter the egg is encapsulated by lamellocytes and destroyed by melanization [22]. We found that the AGFP reporter started to produce a dGFP signal during the encapsulation process (Figure 7B) and it was strongly expressed in fully encapsulated and melanized eggs (Figure 7C). This result revealed that our AGFP reporter system was able to inform us as to the in vivo expression of ADGF-A status in response to a *bona fide* immune challenge within a living animal.

## Discussion

### Generation and verification of reporter

The adenosine deaminase activity of ADGF-A is an important regulator of extra-cellular adenosine in Drosophila larvae [14]. Therefore we decided to make a vital GFP reporter system which would allow us to observe dynamic changes in the ADGF-A expression in vivo.

This work demonstrated that it was possible to use the ‘ends-in’ based method of homologous recombination [23] to specifically and precisely replace a target gene sequence with that of a reporter (or other heterologous sequence). We used this approach to precisely exchange the entire coding-sequence of the *ADGF-A* gene with that of the *dGFP* reporter, leaving intact all the regulatory sequences, including the whole 5′ and 3′ UTRs, of the locus. This allowed us to faithfully reproduce the natural expression pattern of ADGF-A in a manner offering maximal fidelity, especially when compared with more conventional methods of transgenic reporter fly generation that rely on random sites of genomic integration.

Our molecular and phenotypic characterizations of recombination events, coupled with the developmental and the hemocyte-specific expression profiling clearly demonstrated the successful creation of the reporter. Furthermore that it faithfully recapitulates the expression pattern of the endogenous *ADGF-A*. Although newer, and arguably less laborious, strategies to target genes [24] or to tag proteins utilizing novel recombineering strategies [25] are now becoming available, it is important to note that our reporter does represents the first faithful reporter of ADGF-A expression to be published. We anticipate that this will be of use to the wider Drosophila community, especially in light of the absence of anti-ADGF-A antiserum.

Although reporter-derived *AGFP* mRNA was present in all developmental stages (and in similar levels to the endogenous ADGF-A transcripts), we did not detect any GFP protein expression at any developmental stage under normal growth conditions. This may be because the expression levels were too low to be detectable using the available methodology. Additionally in the case of heterozygous animals, the reporter was only expressed from one of the two alleles, thus lowering the potentially detectable expression to ∼50% compared to normal endogenous ADGF-A expression. However, the detectable GFP fluorescence only at the sites of hemocyte aggregation in both AGFP homozygous and heterozygous animals suggests that heterozygosity of the reporter was not the reason. We also used a destabilized version of GFP that is reported to undergo degradation within a couple hours [18]. Whilst this allowed us to observe the important dynamic changes in *ADGF-A* expression, it probably also lowered the overall reporter signal and as a consequence its sensitivity, especially when compared with more stable GFP variants. Therefore we can not conclude that there is no expression of ADGF-A/AGFP at the protein level under normal growth conditions but rather if there is any expression, it is certainly very low.

### Expression of reporter in site of inflammation

In agreement with the previously reported expression of *ADGF-A* mRNA in the hematopoietic organ [12], we found that the *ADGF-A/AGFP* mRNA was quite abundant in hemocytes under normal physiological conditions; typified by circulating and sessile non-activated macrophage-like cells called plasmatocytes. Nevertheless, we rarely observed GFP fluorescence in these cells. However we found that GFP expression was induced in hemocytes during the formation of melanotic capsules, a type of immune response typical of insects [17]. The purpose of melanization is to either isolate and destroy larger foreign bodies that are too big to be phagocytosed or its involvement in the healing of larger wounds. Melanotic capsule formation can be considered a form of inflammatory response since it involves the recruitment and adherence of immune cells (including the macrophage-like plasmatocytes and specialized insect cells called lamellocytes) to large invading objects, as required. Furthermore, both plasmatocytes and lamellocytes expressed the AGFP reporter as they became adhesive and especially in the site of melanotic capsule formation, *i.e.* the site of inflammation.

We used four different ways to induce melanotic capsule formation in larvae. Firstly, in the *adgf-a* mutant, it was induced by a disintegration of endogenous larval tissue, the fat body [14], [26]. Secondly, in the *cactus* mutant, it was induced by constitutive activation of Toll signaling leading to lamellocytes differentiation and the spontaneous aggregation of both plasmatocytes and lamellocytes with consequent melanotic capsule formation [20]. Thirdly, in the *hop^Tum^* mutant, hyperactivation of the JAK/STAT signalling cascade led to melanotic capsule formation [21]. Lastly, melanotic capsule formation was induced by a genuine immune reaction to an egg deposited by a parasitic species of wasp. This last approach demonstrated that the observed AGFP reporter expression did not occur as a consequence of the genetic manipulations used in the other three cases, but rather was indicative of a *bona fide*, in vivo immune response. In all four cases the AGFP reporter was only expressed in the adherent hemocytes of the melanotic capsule, thus indicating that the ADGF-A protein expression is tightly associated with hemocyte function during the immune reaction regardless of the inducing factor and signaling cascades involved.

### Is ADGF-A expression post-transcriptionally regulated?

We did not detect any significant increases in *AGFP* mRNA levels in hemocytes isolated from larvae forming melanotic capsules (and expressing abundantly detectable GFP fluorescence), when compared to hemocytes from *AGFP/+* larvae with no melanotic capsules (and exhibiting a lack of GFP fluorescence). In addition, the *ADGF-A/AGFP* mRNA was quite abundant in these unchallenged wild-type hemocytes, corresponding to ∼10% of the mRNA levels of the ribosomal house-keeping protein gene, *Rp49*. These results suggest the possibility of a post-transcriptional regulative mechanism of ADGF-A/AGFP protein expression. It should be stressed that both 5′ and 3′ UTRs of the *ADGF-A* mRNA were preserved in the *AGFP* reporter mRNA; only the coding sequence was replaced. Therefore, the potential for *adgf-a* gene UTR-mediated post-transcriptional regulation of the GFP reporter sequence exists. Since adenosine is readily transported across the plasma membrane by nucleoside transporters [27], an intriguing possibility may be provided by the potential for a riboswitch mechanism, similar to that present in prokaryotic adenosine deaminase based regulation [28]. Riboswitches have mostly been described as mechanisms of bacterial regulation that enable rapid responses to environmental stimuli. They typically act via the binding of specific ligands (*e.g.* purine molecules) to riboswitch regulatory elements in 5′ UTR of mRNA's, thus prompting or inhibiting their translation. It is therefore possible that when hemocytes find themselves in environments of high concentrations of extra-cellular adenosine, they take the adenosine up and it then binds to a riboswitch within the 5′ UTR of the *ADGF-A* mRNA, that subsequently activates the translation of the ADGF-A protein. Accordingly, we did observe increases in the expression of our AGFP reporter system, when isolated but non-adhered hemocytes were challenged by a dose of exogenous adenosine (data not shown). However we were unable to obtain quantifiable data due to the inherently weak AGFP signal in our system. Furthermore, it is also possible that the activation or adherence of hemocytes also play a role in this regulation that we are unable to yet model. Nevertheless, it will be very important to further explore the mechanism of this post-transcriptional regulation evidently at work in our system and investigate whether a similar mechanism is also operating in mammalian systems.

### What is the role of ADGF/ADA2 in the site of inflammation?

There are at least two ways ADA2 can influence the inflammatory response. Firstly, by its catalytic-independent signaling function and secondly, via regulating adenosine levels through its adenosine deaminase enzymatic activity. It is not known if Drosophila ADGF-A exerts a signaling function similar to human ADA2. This function might be an evolutionary adaptation in vertebrates since the signaling function is associated with adaptive immunity [10] and includes cells that are not present in the innate immunity of Drosophila. Interestingly, insect wounds do not undergo the same burst of cellular proliferation and differentiation that characterizes mammalian wounds healing [29]. However, ADGF-A does shares all the protein domains, including the putative receptor binding domain, with human ADA2 [13] and thus the potential signaling role of ADGF proteins in insects should to be addressed.

The role of ADGF/ADA2 in the site of inflammation is certainly linked to its catalytic activity (the conversion of extra-cellular adenosine to inosine). There are at least two important roles of extra-cellular adenosine during inflammatory response; to mitigate the severity of the potentially harmful response by its anti-inflammatory role and to readjust the energy ‘supply-to-demand’ ratio by stimulating additional blood flow and glucose release from stores.

Changes in the relative amounts of ATP and adenosine form the core of inflammatory response regulation, and act through the purinergic receptors [1]. The release of ATP into the extra-cellular space acts a potently pro-inflammatory, ‘danger-associated molecular pattern’ (DAMP) signal. Such inflammatory processes are associated with a significant increase in the expression of ecto-5′nucleotidase that acts to rapidly convert ATP into adenosine [30]. Furthermore, in vertebrates, adenosine itself is a strongly anti-inflammatory molecule [1] and acts later to down-regulate inflammation. Therefore during acute inflammation, increased extra-cellular adenosine is rapidly metabolized to inosine by adenosine deaminase. Indeed, a close correlation can be observed between inflammation and local increases in adenosine deaminase activity [7], [9]. Our results in Drosophila confirm this correlation in vivo and suggest that adenosine plays a similar role during the inflammatory response of insects as in vertebrates.

However, lowering extra-cellular adenosine levels may have an important function beyond the site of inflammation. Extra-cellular adenosine is traditionally regarded as a local signal due to its usually rapid metabolism; therefore only exerting localized effects on cells/tissues surrounding its site of production. However, studies in the rat model suggest it can act over longer distances in a hormone-like manner [31]. In this rat model, lower-limb ischemia causes the muscular accumulation of both extra-cellular adenosine and inosine that upon reperfusion are rapidly released into the circulating blood. It is these plasma nucleosides that then promote hepatic glucose release and eventual hyperglycemia, via the activation of A3 adenosine receptor on hepatocytes. We recently demonstrated that extra-cellular adenosine can also act as an anti-insulin hormone stimulating a release of glucose from stores in the Drosophila model [26]. Therefore a connection between the work presented here (demonstrating the quite exclusive expression of ADGF-A in sites of inflammation) and our previous study (showing the hyperglycemic effect caused by the deficiency of ADGF-A associated with increased extra-cellular adenosine [26]) could lead to a reappraisal of the roles of extra-cellular adenosine and its regulation by ADGF-A/ADA2. Accordingly, damaged tissues and sites of inflammation generating significant amounts of extra-cellular adenosine, may serve as sites of hormone production that alert an organism towards the appropriate allocation of energy reserves towards mounting a necessary immune reaction. Such a mechanism would need to be under tight control as not to precipitate harmful hyperglycemia and eventually uncontrolled loss of energy reserves. Therefore, once the stimulus for the inflammation is under control, marked by the presence of sufficient activated immune cells at the inflammation site, the signal for energy release should be suppressed. This could explain why ADGF-A protein is only expressed in fully adhered immune cells at site of inflammation *i.e.*, to dampen this important but potentially dangerous signal. The deficiency of ADGF-A protein that causes hyperglycemia and progressive loss of energy reserves in flies [26] illustrates the potential importance of this regulatory circuit. The ability of extra-cellular adenosine to stimulate glucose release over longer distances [31] and the expression of ADA2 in sites of inflammation [7], [9] suggest that similar roles of adenosine and ADA2 in energy allocation could be applicable to mammalian systems.

To better understand the immunomodulatory roles of ATP and adenosine it is important to monitor dynamic changes in the expression of their receptors and the enzymes regulating their in vivo concentrations. This is especially applicable in a time when increasing attention is being paid to the role of adenosine system in inflammation and its involvement in, for example, the pathophysiology of inflammatory bowel diseases [30]. Our work demonstrates that Drosophila could serve as a valuable and convenient model for visualization of such dynamic changes an in vivo context.

### Conclusions

Our work reports the creation of a functional in vivo expression reporter for ADGF-A using precise gene replacement homologous recombination procedures. Results of this work confirm the expression of ADGF/ADA2 enzymes in the site of inflammation by showing that ADGF-A expression occurs specifically in the adhered immune cells of such sites in Drosophila. This supports the view that the inflammatory response and its regulation are evolutionary ancient and Drosophila and mammalian systems share common mechanistic features. Our model also suggests that the expression of adenosine deaminase during inflammation response might be regulated at post-transcriptional level, therefore potentially providing a means of rapid responding regulation. Our previously uncharacterised observations of ADGF-A expression highlight its potential regulatory role in energy allocation stimulated by extra-cellular adenosine.

## Supporting Information

File S1 Details of design and production of AGFP reporter by homologous recombination.
(PDF)
