# Supplementary material for: Expression of Drosophila Adenosine Deaminase in Immune Cells during Inflammatory Response
Source: PLoS One. 2011 Mar 11;6(3):e17741. doi: 10.1371/journal.pone.0017741 (PMC3055890; doi:10.1371/journal.pone.0017741)
Supplement: File S1 — Details of design and production of AGFP reporter by homologous recombination. (PDF) [file pone.0017741.s001.pdf]

Expression of Drosophila adenosine deaminase in immune cells during inflammatory response

Milena Novakova[1] and Tomas Dolezal[1,2]

[1]Department of Molecular Biology, Faculty of Science, University of South Bohemia, Ceske Budejovice, Czech Republic

[2]Corresponding author: Faculty of Science, University of South Bohemia, Branisovska 31, 37005 Ceske Budejovice, Czech Republic. Phone: +420-387772229 E-mail: tomas.dolezal@prf.jcu.cz

SUPPORTING INFORMATION S1

Details of design and production of AGFP reporter by homologous recombination

Materials and methods

Design of homologous recombination construct and cloning: We used Drosophila *yw* strain genomic DNA to amplify three fragments. Using primer F1 5´-gcg gcc gcc atg aaa tcc aca tca agg gg-3´ (introducing *Not*I site to 5´ terminus) and primer R1 5´-tgc tca cca tga tga gcg gta gat ttc gtt ag-3´ (introducing 9 bases from the *dGFP* coding sequence to 5´ terminus) we amplified a 4.3-kb long fragment 1 containing the *ADGF-A2* gene and promotor sequence of the *ADGF-A* gene. With primer F3new 5´-gct ag c acg gtt ggt ggc ag-3´ (introducing *Nhe*I site to 5´ terminus) and primer R3new2 5´- ggg ccc ata tct ctt cct tct tga cgg-3´ (introducing *Apa*I site to 5´ terminus) we amplified a 2.2-kb fragment 3 including a short part of the *ADGF-B* gene and the upstream sequence. A 6-kb fragment 4 was amplified with primer F4 5´- ggg ccc cgt tgg gcg cca ttt tgt gc-3´ (introducing *Apa*I site to 5´ terminus) and primer R4new 5´-ggt acc gtc gtc ctg ttc ctg ttt cc-3´ (introducing *Kpn*I site to 5´ terminus) covering the *ADGF-B* gene and the downstream and upstream sequences.
From plasmid Casper4 containing the *dGFP* sequence (provided by Tamas Lukacsovich) we amplified with primer F2 5´-acc gct cat cat ggt gag caa ggg cga gg-3´ (introducing 10 bases from the *ADGF-A* promoter to 5´ terminus) and primer R2new 5´-gct ag c tac aca ttg atc cta gc-3´ (introducing 5 bases from the *ADGF-A* terminator and *Nhe*I site to 5´terminus) a 861-bp fragment 2 containing *dGFP* coding sequence.
Fragments 1 and 2 were amplified using standard PCR and then combined in 2 steps by recombinant PCR. In the first step 5 µl of each fragment from unpurified PCR reactions were added to 30-µl PCR mixture without primers and annealed for five PCR cycles. In the second step 40 µl of unpurified PCR mixture from the first step was added to 60 µl of PCR mixture with the outer primers F1 and R2new and combined fragments were amplified for additional five cycles. A 5.2-kb recombinant fragment was then purified from agarose gel and cloned into the pGEM-T Easy vector (Promega).
Fragments 3 and 4 were also cloned into pGEM-T Easy. Fragment 3 was combined using inserted restriction sites to recombinant fragment and together re-cloned from pGEM-T Easy to pBLUESCRIPT II SK+ vector. An I-*Sce*I site was inserted in to the fragment 4 by annealing oligonucleotides Sce-ApaI 5´-cta ggg ata aca ggg taa t-3´ and Sce-REM 5´- cta gat tac cct gtt atc cct agg gcc-3´ (introducing *Apa*I and *Nhe*I site compatible overhangs) and ligating into *Apa*I and *Nhe*I sites of fragment 4, destroying the *Nhe*I site. Introduced I-*Sce*I sequence replaced ~1.9 kb of the 5'-end sequence originally amplified in fragment 4 (combination with the 3' end of fragment 3 deleted ~1.4 kb region in the *ADGF-B* gene in the final). Fragment 4 with I-*Sce*I site was then transferred from pGEM-T Easy to the construct in pBLUESCRIPT II SK+ using *Apa*I and *Kpn*I sites. The assembled construct was finally transferred from

pBLUESCRIPT II SK+ to pTV2 plasmid (provided by Yikang Rong and Kent Golic) using *Not*I and *Kpn*I sites (Figure S1A). The *dGFP* sequence and I-*Sce*I site was verified by sequencing and restriction analysis.

Fly stocks: In the first step of homologous recombination (HR) we used *yw; P[ry+, 70FLP]4 P[v+, 70I-SceI]2B Sco/S(2)Cyo* and *w[1118]; P[ry+, 70FLP]10* (constitutively active FLP recombinase, homozygous on the second chromosome) fly stocks. In the second step of HR we used *v P[70I-CreI, v+]2A; ry/TM3 Ser* stock. All stocks were provided by Yikang Rong and Kent Golic. The stocks *yw; Xa/Cyo; MKRS* and *w; TM3 Sb Ser/TM6B* were used for the mapping of insertions and to establish the recombinant lines.
DNA construct for HR was injected in to the *yw* embryos using the modified P-element-mediated transformation procedure (PARK and LIM 1995). To induce HR we used the rapid targeting scheme (RONG and GOLIC 2001), described in details in DOLEZAL *et al*. (2003).

Molecular analysis of the targeted events: The first step of HR was analyzed using standard methods of DNA isolation, PCR analysis, restriction and southern blot analysis. DNA from all tested lines (homozygous or heterozygous adults) were digested with restriction enzyme *Xho*I and hybridized with a 1.6-kb ADGF-A RNA-probe or with a 864-bp dGFP RNA-probe labeled by DIG (Maxiscript *in vitro* transcription kit, Ambion Austin, TX) (Figure S1B). After the second step of HR, selected lines were tested for the presence of *dGFP*. Genomic DNA was isolated from heterozygous adults and digested by *Xho*I and *Kpn*I restriction enzymes. Southern blot was hybridized with the dGFP RNA-probe. Recombination events were further verified by PCR analysis and sequencing.

Results and commentaries

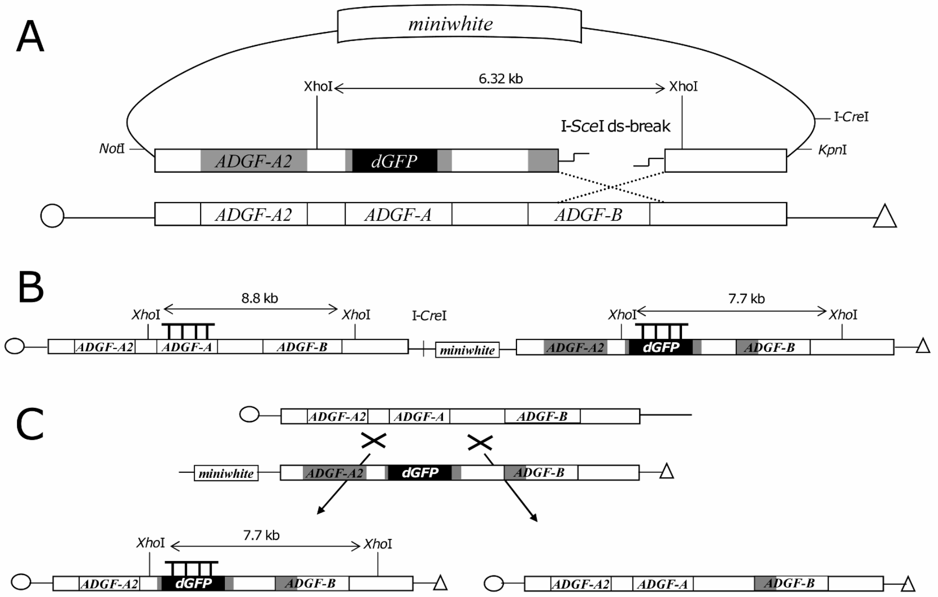

Figure S1. Design of targeting construct and two steps of homologous recombination. (A) The first step of homologous recombination and construct design. The *ADGF-A* coding sequence (from start to stop codon containing two short introns) was exchanged by coding sequence of *dGFP*. I-*Sce*I site replaced ~1.4-kb distal region of the *ADGF-B* gene starting 2.2 kb downstream of the *dGFP* stop codon. 4-kb long homologous sequence distal from *ADGF-B* was placed on the other side of I-*Sce*I site. The whole homologous region with *dGFP* and I-*Sce*I site was cloned into the pTV2 vector containg *miniwhite* marker. I-*Sce*I-induced double-strand break in donor DNA (top) stimulates recombination with the homologous sequence on chromosome III (drawn as rectangle). (B) The structure of an expected duplication after the first step of HR produced by recombination between the donor DNA and the target locus. The centromeric side (represented by circle) of the duplication contains wild-type gene arrangement, on the telomeric side (represented by triangle) the *ADGF-A* gene is replaced by the *dGFP* sequence. (C) The second step of HR. I-*Cre*I is used to produce DSB between the duplicated regions resulting in a reduction event. Recombination occurring upstream of the *ADGF-A/dGFP* sequences results in the chromosome carrying *dGFP* sequence (left). Recombination downstream of the *ADGF-A/dGFP* sequences leads to wild-type gene arrangement (right). Positions of the *Xho*I restriction sites and the corresponding sizes of the fragments on southern blot are indicated above the sequences; binding of the ADGF-A and dGFP probes are marked by comb-like structures. Grey boxes represent gene sequences including untranslated regions originated from the donor construct; white boxes indicate chromosomal gene sequences; the black box represents *dGFP* coding sequence and box with *miniwhite* represents an eye-color marker used for screening.

For the first step of homologous recombination (HR) we used a donor line (M2) with the P element targeting construct inserted on the second chromosome to avoid the complication with the remaining FRT sequence and P-element repeats (targeted sequence is on chromosome III). We recovered 15 potential recombinant lines from total 241 screened vials.

In 7 of those lines (Table S1) the events were mapped to the third chromosome where the *ADGF-A* gene is located, while remaining 8 events were mapped to the second chromosome and thus labeled as non-targeted events and discarded. All targeted lines were tested by southern blot and PCR analysis for the changes in the targeted region (Figure S2). We digested genomic DNA by an *Xho*I restriction enzyme and used probes for the *ADGF-A* and the *dGFP* sequences. This allowed us to distinguish between the centromeric side of the duplication with 8.8 kb if the *ADGF-B* sequence was properly repaired (see further), the telomeric side of the duplication with 7.7 kb (with the *dGFP* replacement and again the proper *ADGF-B* repair) and the original or non-targeted construct insertion with 6.3 kb (i.e. with the *dGFP* sequence and the deletion in the *ADGF-B* region; see Figure S1 for details).

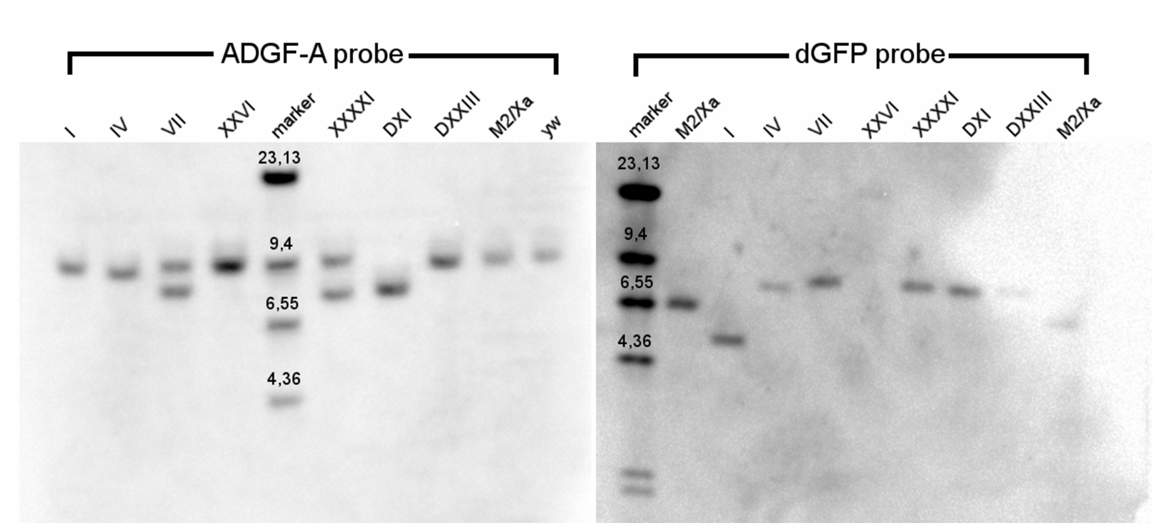

Figure S2. Southern blot analysis of lines with targeted events after the first step of homologous recombination. Genomic DNA was isolated from *yw* control flies, M2/Xa line with the original insertion of the donor construct and from heterozygous (lines I, IV, VII, XXVI and XXXXI) and homozygous (lines DXI and DXXIII) adult flies with targeted events. DNA was digested with *Xho*I restriction enzyme and hybridized with probes specific for the *ADGF-A* or the *dGFP* sequence (see also scheme on Figure S1 for the fragment sizes). Sizes in kb are depicted above each band of the DIG-labeled marker (Roche, Mannheim, Germany).

Four of the tested lines detected by the *ADGF-A* probe (labeled as I, IV, XXVI and DXXIII) produced the expected fragments for centromeric side of the duplication. Three duplications (VII, XXXXI and DXI) produced approx. 1.5-kb shorter fragments on this side of the duplication indicating deletions in the vicinity of the original I-*Sce*I site (in the cases of the VII and XXXXI lines the DNA originated from heterozygous flies explaining the presence of the additional wild type fragments). The I-*Sce*I site in the donor construct was accompanied by a deletion in the *ADGF-B* region compared to the homologous wild-type sequence (see Material and methods and Figure S1A for details). Therefore we expected that in some cases the repair of this gap during HR process might be imperfect. An additional PCR and sequencing analysis of the XXXXI and DXI lines confirmed deletions in the *ADGF-B* region. In the case of DXI line we detected a deletion of a 1.2-kb region in the *ADGF-B* gene and downstream sequence which was shorter and shifted compared to the original deletion in the donor construct. A 1.4-kb deletion found in XXXXI line was precisely the same size and location as in the original donor construct including the I-*Sce*I site in this duplication. The

deletion occurrence in the vicinity of double strand break had been previously discussed by RONG and GOLIC (2000) and EGLI *et al*. (2003). In the case of the DXI duplication, the deleted region formed probably as a result of non homologous joining of sequence placed on the right side of I-*Sce*I in donor construct (Figure S1A) to the *ADGF-B* targeted region during the crossing-over mechanism. The other side of the duplication with *dGFP* was repaired by re-synthesis using chromosomal sequence as a template to the wild-type state. Possible explanation for a character of the XXXXI duplication with preserved I-*Sce*I site is an existence of the second donor construct during G2 phase of the cell cycle. Events explainable by the presence of the second donor construct during recombination process were previously documented (RONG and GOLIC 2000; DOLEZAL *et al.* 2003). LANKENAU *et al*. (2003) also found such events and offered two possible mechanism for I-*Sce*I site preservation: dimerization of donor construct with only one double strand break and subsequent resection of the ends and classical double strand break repair or using BIR (break-induced recombination) model where the intact I-*Sce*I site is explained by an endogenous double strand break in the target gene but not in a donor construct. In the case of the XXXXI duplication the double strand break would occur downstream of the *ADGF-B* gene that match with donor construct sequence (Figure S1A). Detailed characterization of the duplication event VII was not performed.

For the second step of HR we chose five lines - IV, VII, XXXXI, DXI, DXXIII - carrying duplications in the *ADGF-A* region and crossed them with the line carrying the CreI endonuclease. The progeny was heat-shocked early in their development to induce CreI expression resulting in a loss of the *miniwhite* marker (Figure S1C). Selected white-eyed flies were crossed individually to establish balanced stocks. After I-*Cre*I mediated reduction event, we recovered totally 53 lines (Table S1); 14 of them were subjected for further southern blot and PCR analysis. Genomic DNA was isolated from heterozygous lines and digested by two different restriction enzymes *Xho*I and *Kpn*I; only *Xho*I is shown, *Kpn*I confirmed results with *Xho*I in all cases. We used *Kpn*I because the site for this enzyme was localized outside the boundaries of homology with the donor construct and thus confirmed that all tested events were targeted.

Mutations causing an early lethality most likely occurred in lines 9-3, 47-3 and 32 during the recombination processes. The homozygous viability of reductions 23 and 27 originating from the same duplication as line 32 as well as the viability of the original duplication stock DXI indicate that some changes appeared during the second recombination step leading to reduction 32. On the other hand, mutations leading to early lethality in lines 9-3 and 47-3 most likely occurred already during the first step of HR. This is supported by two observations. First, it is the absence of homozygotes in the original duplication stock IV. Second, it is homozygous lethality in line 8-4 which originated from the same duplication as lines 9-3 and 47-3 but the *dGFP* sequence was not detected in this line and thus we expected that the wild-type gene arrangement would be associated with normal viability. In comparison, reduction 74-3 from a different duplication where the wild-type gene arrangement was also restored expressed the wild-type phenotype.

TABLE S1

SUMMARY OF TARGETED EVENTS AND THEIR MOLECULAR AND PHENOTYPIC CHARACTERIZATION

Dupli-cation	Southern blot analysis of duplications	Total number of isolated reductions	Reductions analyzed by Southern blot	Southern blot results	Phenotype of homozygotes
**I**	GFP side ~2.5 kb shorter	0	0	-	-
			8-4	no GFP signal	early lethality
			9-3	expected size	early lethality
**IV**	duplication as expected	15	47-3	expected size	early lethality
			49-2	expected size	*adgf-a* mutant
			57	expected size	*adgf-a* mutant
**VII**	ADGF-A side ~1.5 kb shorter	17	74-3	no GFP signal	wild type
**XXVI**	GFP side - no signal	0	0	-	
			72	expected size	*adgf-a* mutant
			77	expected size	*adgf-a* mutant
**XXXXI**	ADGF-A side ~1.5 kb shorter	6	85	expected size	*adgf-a* mutant
			23	expected size	*adgf-a* mutant
			27	expected size	*adgf-a* mutant
**DXI**	ADGF-A side ~1.5 kb shorter	12	32	expected size	early lethality
			33	expected size	*adgf-a* mutant
**DXXIII**	duplication as expected	3	37	expected size	*adgf-a* mutant
**total 7 / 241**		**total 53**	**total 14**		

References

Dolezal T, Gazi M, Zurovec M, Bryant PJ (2003) Genetic analysis of the ADGF multigene family by homologous recombination and gene conversion in Drosophila. Genetics 165: 653–666.

Egli D, Selvaraj A, Yepiskoposyan H, Zhang B, Hafen E et al. (2003) Knockout of ´metal-responsive transcription factor´MTF-1 in Drosophila by homologous recombination reveals its central role in heavy metal homeostasis. EMBO J. 22: 100–108.

Lankenau S, Barnickel T, Marhold J, Lyko F, Mechler M et al. (2003) Knockout Targeting of the Drosophila Nap1 Gene and Examination of DNA Repair Tracts in the Recombination Products. Genetics 163: 611–623.

Park S, Lim JK (1995) A microinjection technique for ethanol treated eggs and a mating scheme for detection of germ line transformants. Dros. Inf. Serv. 76: 187–189.

Rong YS, Golic KG (2000) Gene targeting by homologous recombination in Drosophila. Science 288: 2013–2018.

Rong YS and Golic KG (2001) A targeted gene knockout in Drosophila. Genetics 157: 1307–1312.
